# Do ask, do tell: improving health outcomes for sexual and gender minorities with cancer

**DOI:** 10.1093/jncics/pkad075

**Published:** 2023-10-19

**Authors:** Daniel R Dickstein, Eric Chen, Nicholas G Zaorsky, Karen Hoffman, Paul Nguyen

**Affiliations:** Department of Radiation Oncology, Icahn School of Medicine at Mount Sinai, New York City, NY, USA; Department of Radiation Oncology, University Hospitals Seidman Cancer Center, Case Western Reserve University, Cleveland, OH, USA; Department of Radiation Oncology, University Hospitals Seidman Cancer Center, Case Western Reserve University, Cleveland, OH, USA; Department of Radiation Oncology, The University of Texas MD Anderson Cancer Center, Houston, TX, USA; Department of Radiation Oncology, Dana-Farber Brigham Cancer Center, Boston, MA, USA

As clinicians, scientists, and researchers, we are responsible for creating an equitable health-care system for all patients, including sexual and gender minority patients. These patients represent approximately 7.2% of the US population (1) and an estimated 141 000 newly diagnosed cancer cases annually (2). Yet, there are limited studies addressing the unique needs of sexual and gender minority patients with cancer, an issue that continues as the cancer health-care community fails to collect sexual orientation and gender identity data in clinical trials and in clinical practice (3). In their cross-sectional analysis, Yang et al. (1) show that the systematic collection of sexual orientation and gender identity data are feasible and important for understanding differences in the health-care needs of sexual and gender minority and non–sexual and gender minority people with cancer.

In their study, Yang and researchers determine that 2.7% of their cancer cohort self-identify as a sexual and gender minority (1). The authors acknowledge that the study’s sexual and gender minority population is smaller than both the hospital catchment area sexual and gender minority population (4.1%) and the US sexual and gender minority population (7.2%). Nevertheless, the authors should be commended for obtaining sexual orientation and gender identity information for more than 50 000 patients with cancer, 1 of the largest cohorts with sexual orientation and gender identity information. Notably, the catchment area for this study, spanning west central and central Florida, is socially conservative (4,5). Cancer care professionals working in socially conservative areas may feel apprehensive about asking patients about their sexual orientation and gender identity. Similarly, sexual and gender minority individuals—in theory—may be less willing to disclose their true orientation or identity to their health-care professionals in more conservative regions (5). The success of this study should inspire clinicians in all geographic regions to collect sexual orientation and gender identity demographic data.

Despite their study having a relatively small percentage of patients indicating that they are sexual and gender minority individuals, Yang and researchers (1) demonstrate that many patients are in fact comfortable answering sexual orientation and gender identity questions (3,6). The Institute of Medicine, American College of Physicians, and the American Society of Clinical Oncology (ASCO), in 2010, 2015, and 2017, respectively, acknowledged the health-care disparities that sexual and gender minority individuals experience and called for the systematic collection of sexual orientation and gender identity data; yet, health-care professionals still fail to ask about sexual orientation and gender identity. An analysis of 764 cancer-specific phase 3 randomized controlled trials revealed that cancer-related clinical trials do not collect sexual orientation and gender identity data (7). Furthermore, a survey of American Society of Clinical Oncology members showed that fewer than half of oncologists ask their patients about sexual orientation and gender identity; moreover, some consider collecting sexual orientation (17%) or gender identity (12%) data unimportant (8). Respondents cited personal comfort as well as institutional culture, lack of support, and minimal resources as barriers to the collection of sexual orientation and gender identity data (8). Yang et al. note that the National Cancer Institute sexual orientation and gender identity data-collection initiative was launched less than a year ago and provided funding to only 14 of 71 National Cancer Institute Designated Cancer Centers for this data collection (1,9). To begin addressing the long-standing health inequities for sexual and gender minority individuals with cancer (10), the systematic collection of sexual orientation and gender identity information across all practices is necessary, and institutional support and resources are vital to its success (8).

Through the collection of sexual orientation and gender identity data, Yang et al. (1) illustrate that researchers can indeed identify the unique health needs within this community. They demonstrate that sexual and gender minority individuals with cancer experience more psychosocial distress than non–sexual and gender minority individuals with cancer (1). Sexual and gender minority individuals with cancer are less likely to have social support, such as romantic partners, family members, or friends, during treatment than non–sexual and gender minority individuals (95.7% vs 97.3%, *P *= .001). A lack of social support is associated with worse disease and survival outcomes in patients with cancer, potentially through increased treatment interruptions and worse toxicity management (11). Furthermore, it has been established that social support for sexual and gender minority individuals may not mirror that of non–sexual and gender minority individuals (12,13). Previous reports noted that the social support (eg, friends, partners) for sexual and gender minority individuals with cancer are not provided the same resources, engaged in the same discussions, or treated as fairly as family members of non–sexual and gender minority individuals with cancer, which the authors attribute to social support for sexual and gender minority individuals appearing nontraditional compared with that of non–sexual and gender minority individuals (12). Sexual and gender minority individuals may be more likely to identify a chosen family member as opposed to a biological family member, and research has illustrated that these chosen family cancer caregivers experience similar difficulties and emotions, such as distress, as “traditional” cancer caregivers (13). Cancer health care professionals must be aware of the unique psychosocial needs, risks, and support structures of sexual and gender minority individuals with cancer to provide equitable care.

In addition to a difference in social support, Yang and colleagues (1) establish that sexual and gender minority individuals with cancer are more likely to live alone than non–sexual and gender minority individuals with cancer (19.2% vs 13.8%, *P *< .001)—an approximate 40% relative increase in the percentage of sexual and gender minority individuals with cancer living alone. This difference may have multiple reasons, such as fewer sexual and gender minority individuals subscribing to heteronormative expectations of family formation (14), social marginalization (15), or the lasting impacts of the HIV and AIDS epidemic on older sexual and gender minority individuals (16). Consistently, other studies have suggested that sexual and gender minority older adults (≥65 years of age) are more than twice as likely to live alone than non–sexual and gender minority older adults, which is especially important to note because older adults also have additional psychosocial needs (17). In the current study by Yang and colleagues, it is surprising that 73% of sexual and gender minority individuals are aged 20 to 64 years—vs 53% of non–sexual and gender minority individuals—and there are not more older adults in the sexual and gender minority cohort to drive differences in mental health, social support, and living alone (1). The unexpected finding that this younger population of sexual and gender minority individuals have worse mental health, less social support, and are living alone is worrisome, and further analyses by age may be valuable (1). These differences may be important because loneliness has been associated with other health conditions, including depression, substance use disorders, and pain, all of which can affect cancer care and resulting outcomes (18,19).

Consistently, Yang et al. highlight that sexual and gender minority individuals with cancer experience more bodily pain than non–sexual and gender minority individuals with cancer, which could be attributed to the relative increase in loneliness. Sexual and gender minority individuals with cancer scored an average of 3.3 points lower on the bodily pain subscale of the physical component summary score, which ranges from a 0 to 100 points (a lower subscale score indicates worse outcomes—that is, more pain) (1), than non–sexual and gender minority individuals (mean score = 65.5 vs 68.8). Despite the mean scores being relatively similar, 3.3 is above the minimum clinically important difference of 3 for this subscale (20), as validated in the general US population (18). The meaningful difference makes sense given that other studies have illustrated that cancer survivors who experience loneliness are more likely to experience bodily pain (21), and brain imaging has revealed that people who feel lonely, vulnerable, and excluded have similar brain activity to people who experience physical pain (22). Research has suggested that the observed increase in physical pain associated with feelings of loneliness is through an inflammatory response to feelings of rejection and vulnerability (18), and it has been found that acetaminophen can help relieve both physical and mental pain (23,24). Additionally, a research team discovered that sexual minority men (ie, gay and bisexual men) who were not open about their sexual orientation were at greater risk for acquiring infections, developing chronic diseases, and having a shorter lifespan than sexual minority men who were open about their sexual orientation (25,26). It is essential for cancer care clinicians to understand the connection between physical pain and emotional difficulties in the context of sexual and gender minority individuals with cancer because the fear of revealing their sexual and gender minority identity, the emptiness of living alone, and the anxiety of a cancer diagnosis can all contribute to and exacerbate the physical pain both from cancer and from its treatments (6). By simply asking a patient’s sexual orientation and gender identity, a physician may be able to help alleviate the patient’s sense of isolation, anxiety, and pain. Future studies are warranted to further elucidate the impact of loneliness and isolation on physical functioning for patients with cancer, especially in this vulnerable population.

We commend the authors for publishing this study and illustrating that the widespread collection of sexual orientation and gender identity data is not only feasible but essential to uncover health disparities affecting sexual and gender minority individuals with cancer, including differences in psychosocial distress. Through their systematic efforts and institutional support, challenges to the psychosocial needs of sexual and gender minority individuals with cancer have been identified, and future interventions may in turn be proposed, tested, and implemented.

## Data Availability

No new data were generated or analyzed for this editorial.
